# Decreased cardiac excitability secondary to reduction of sodium current may be a significant contributor to reduced contractility in a rat model of sepsis

**DOI:** 10.1186/cc13800

**Published:** 2014-03-26

**Authors:** Andrew Koesters, Kathrin L Engisch, Mark M Rich

**Affiliations:** 1 Department of Neuroscience, Cell Biology, and Physiology, Wright State University, Dayton, OH 45435, USA

## Abstract

**Introduction:**

Multisystem organ failure remains a poorly understood complication of sepsis. During sepsis, reduced excitability contributes to organ failure of skeletal muscle, nerves and the spinal cord. The goal of this study was to determine whether reduced excitability might also contribute to cardiac failure during sepsis.

**Methods:**

Wistar rats were made septic by cecal ligation and puncture. One day later, action potentials were recorded from beating left ventricular papillary muscle *ex vivo* by impaling myocytes with sharp microelectrodes.

**Results:**

In cardiac papillary muscle from septic rats, action potential amplitude and rate of rise were reduced, while threshold was elevated. These changes in action potential properties suggest sepsis selectively reduces sodium current. To determine the effects of selective reduction in sodium current, we applied tetrodotoxin to papillary muscle from healthy rats and found reduction in action potential amplitude and rate of rise, as well as elevation of threshold. The changes were similar to those triggered by sepsis. Blocking calcium current using nifedipine did not mimic action potential changes induced by sepsis. Contractility of healthy papillary muscle was reduced to 40% of normal following partial block of sodium current by tetrodotoxin, close to the low contractility of septic papillary muscle, which was 30% of normal.

**Conclusions:**

Our data suggest cardiac excitability is reduced during sepsis in rats. The reduction in excitability appears to be primarily due to reduction of sodium current. The reduction in sodium current may be sufficient to explain most of the reduction in cardiac contractility during sepsis.

## Introduction

Multisystem organ failure is a devastating complication of critical illness. The mechanisms underlying multisystem organ failure remain mysterious with no unifying hypothesis to explain its occurrence. We and others have shown there is reduction in excitability of skeletal muscle in both patients and animal models of critical illness [1-5]. More recently, we and others have also shown there is reduction in excitability of peripheral nerve in both patients and an animal model of sepsis [6,7]. Additionally, we recently demonstrated reduction in motor neuron excitability within the central nervous system in septic rats [8]. These studies suggest reduced excitability occurs within multiple electrically active tissues during critical illness.

In rat models of critical illness it has been possible to determine the defect underlying reduced excitability. In both peripheral nerve and skeletal muscle the defect appears to be reduction in Na current due to altered voltage dependence of inactivation of channels [7,9-12]. These studies raise the possibility that an acquired Na channelopathy may be a common consequence of critical illness that occurs in multiple electrically active tissues.

While it is known that severe sepsis causes cardiac dysfunction [13-16], there have been no studies directly examining excitability of the heart during critical illness. We previously performed a retrospective study of electrocardiogram (ECG) changes in patients during and following recovery from sepsis and found reversible reductions in QRS amplitude as well as increases in QRS duration [17]. These changes are consistent with reduction of cardiac excitability, but other interpretations such as reduction in QRS amplitude secondary to anasarca cannot be ruled out [18,19]. Here, we used a rat model of sepsis to directly measure changes in cardiac excitability during sepsis. The data suggest that during sepsis cardiac excitability is decreased due to a reduction in Na current, and reduction in Na current alone can reduce contractility.

## Materials and methods

### Induction of sepsis

All animal protocols were approved by the Institutional Animal Care and Use Committee at Wright State University (Animal Assurance number A3632-01). Sepsis was induced in isoflurane-anesthetized adult female Wistar rats (250 to 300 grams) by cecal ligation and puncture as described previously [7]. For continuous relief of pain, an Alzet 2 mL osmotic pump (Durect Corp., Cupertino, CA, USA) that delivered 20 μg/kg/hr of hydromorphone was inserted into the abdomen prior to closing the incision. At the end of surgery, rats were given a single dose of buprenorphine (0.12 mg/kg) subcutaneously for pain relief until the hydromorphone took effect. Rats were studied one day after induction of sepsis as we have found previously that one day is sufficient to induce reduction of motoneuron excitability [8]. A subset of rats did not survive 24 hours after induction of sepsis and only rats exhibiting severe signs of sepsis 24 hours after cecal ligation and puncture were euthanized and used for experiments. Rats were determined to be severely septic and killed for experiments when they exhibited piloerection of the pelt, a hunched stance, little to no spontaneous movement, and had a blunted response to touching and handling.

### Preparation of left ventricular papillary muscle

One day after cecal ligation and puncture, rats were euthanized via CO_2_ asphyxiation, and hearts were removed and placed into a Ca^2+^-free phosphate-buffered solution to wash away excess blood. The papillary muscle was removed and pinned at each end; then perfused with a low-Ca^2+^ solution containing (in mM): NaCl (118), KCl (3.5), MgSO_4_ (0.7), NaH_2_PO_4_ (1.7), NaHCO_3_ (26.2), dextrose (5.5), CaCl_2_ (0.375), and 2,3-butanedione monoxime (BDM) (Sigma-Aldrich, St Louis, MO, USA) 5 mM); then bubbled with 95% O_2_ and 5% CO_2_. Bath temperature was 22°C, pH was 7.4. This temperature was chosen as we found that excitability of papillary muscle was difficult to maintain over time at warmer temperatures. 4-Di-2ASP (DiASP, Molecular Probes, Eugene, OR, USA) (10 μM) was added to the bathed muscle for 3 to 4 minutes to allow for visualization of cardiac muscle using epifluorescence. We use this dose of 4-Di-2ASP routinely for imaging of skeletal muscle fibers and neuromuscular junctions during recordings of muscle fiber action potentials [5,20]. The papillary muscle was perfused with low-Ca^2+^ solution containing BDM for 15 minutes, followed by 15 minutes of perfusion with low-Ca^2+^ solution without BDM. The inclusion of BDM greatly improves viability of preparations [21,22]. Following low-Ca perfusion, we perfused for 10 minutes with solution containing normal extracellular Ca^2+^ (1.5 mM). Muscles were then paced for 1 hour using 1 ms current pulses generated by a stimulus isolation unit (model A360 WPI Inc., Sarsota, FL, USA) and delivered via a bipolar extracellular concentric electrode (FHC Inc., Bowdoin, ME, USA) with a stimulation rate of 1 Hz. The electrode was placed near the end of the muscle and the stimulus was set at 1.5x the minimum strength necessary to elicit maximal muscle contraction. Muscle twitch was monitored over the next 1-hour period and stimulus strength was gradually decreased as muscle excitability recovered [23]. As BDM decreases cardiac contractility [24], papillary muscles were perfused in solution lacking BDM for 1.5 hours prior to force measurements. Using the above protocol, excitability of papillary muscles was stable for greater than 2 hours at 22°C.

### Intracellular recording from cardiomyocytes

After pacing for 1 hour, muscles were imaged using an upright epifluorescence microscope (Leica Microsystems, Bannockburn, IL, USA). Myocytes were impaled using sharp electrodes filled with 3 M KCl solution and containing sulforhodamine (1 mM) to allow for visualization of the tip. Tip resistances were between 15 to 35 MΩ, and capacitance compensation was optimized for each electrode prior to recording. Myocytes were impaled with a single electrode while pacing of the papillary muscle continued, and action potentials were recorded using a Gene Clamp 500B amplifier (Axon Instruments/Molecular Devices, Sunnyvale, CA, USA), and a custom software package (M. Pinter, Emory University). In a subset of fibers, pacing was briefly turned off and membrane time constant was measured by impaling single myocytes with two sharp electrodes and measuring the membrane response to a 10 ms injection of hyperpolarizing current. Pacing was restarted after each recording and continued for 2 to 3 minutes before impaling the next fiber.

### Effects of tetrodotoxin and nifedipine on action potentials and contractility in healthy muscle

After recording action potentials from four to six fibers, tetrodotoxin (TTX, 5 μM, Tocris Cookson, Ellisville, MO, USA), or nifedipine (10 μM, Sigma-Aldrich) was added to the perfusate. After 7 to 10 minutes, action potentials were recorded from an additional five to eight fibers. The papillary muscle from each rat served as its own control. To determine the effect of TTX and nifedipine on contractility, the papillary muscle was attached to a linear motor with force transducer (Model 305B-LR, Aurora Scientific, Aurora, ON, Canada) on one end so that force could be recorded during pacing. Muscle length was measured in the heart prior to removal, and *ex vivo* length was adjusted by a motor on the force transducer to equal the *in situ* length. After 1 hour of pacing, force generated was recorded at several lengths to identify the length that produced maximal force. Once length was optimized, force was recorded; then TTX (5 μM) was added to the perfusate and allowed to equilibrate for 15 minutes. Force was recorded; TTX was then washed out for 15 minutes, and force was again recorded. Nifedipine (10 μM) was then added to the perfusate and allowed to equilibrate for 15 minutes. Force was recorded; nifedipine was then washed out for 1 to 2 hours, and final force record was recorded. Measurement of papillary muscle force was performed using Spike5 software (Cambridge Electronic Design, Cambridge, UK).

### Data analysis and sample size

Action potential data were analyzed using OriginPro 8 (OriginLab Corp., Northampton, MA, USA). Fibers with resting potentials more depolarized than −72 mV were excluded from analysis. Less than 10% of fibers were discarded in healthy and septic rats. Action potential rate of rise (dV/dt) was determined by taking the derivative of voltage with respect to time using Origin software. Action potential threshold was defined as the voltage at which dV/dt was >10 mV/ms. In each papillary muscle, action potentials were measured from at least four fibers. An average value was then obtained for each parameter for each papillary muscle. Animal average data were tested for normal distribution and parameters were compared in control vs. septic rats using the papillary muscle means for an unpaired Student’s *t* test, with n as the number of rats studied. For the study of sepsis versus control at least 80 muscle fibers were recorded from for each condition. For the TTX and nifedipine studies, each rat served as its own control and a paired Student’s *t* test was used. A parametric test was used as the larger control data set suggested the values studied were normally distributed. In these studies at least 14 muscle fibers were recorded from for each condition.

## Results

**Figure 1:**
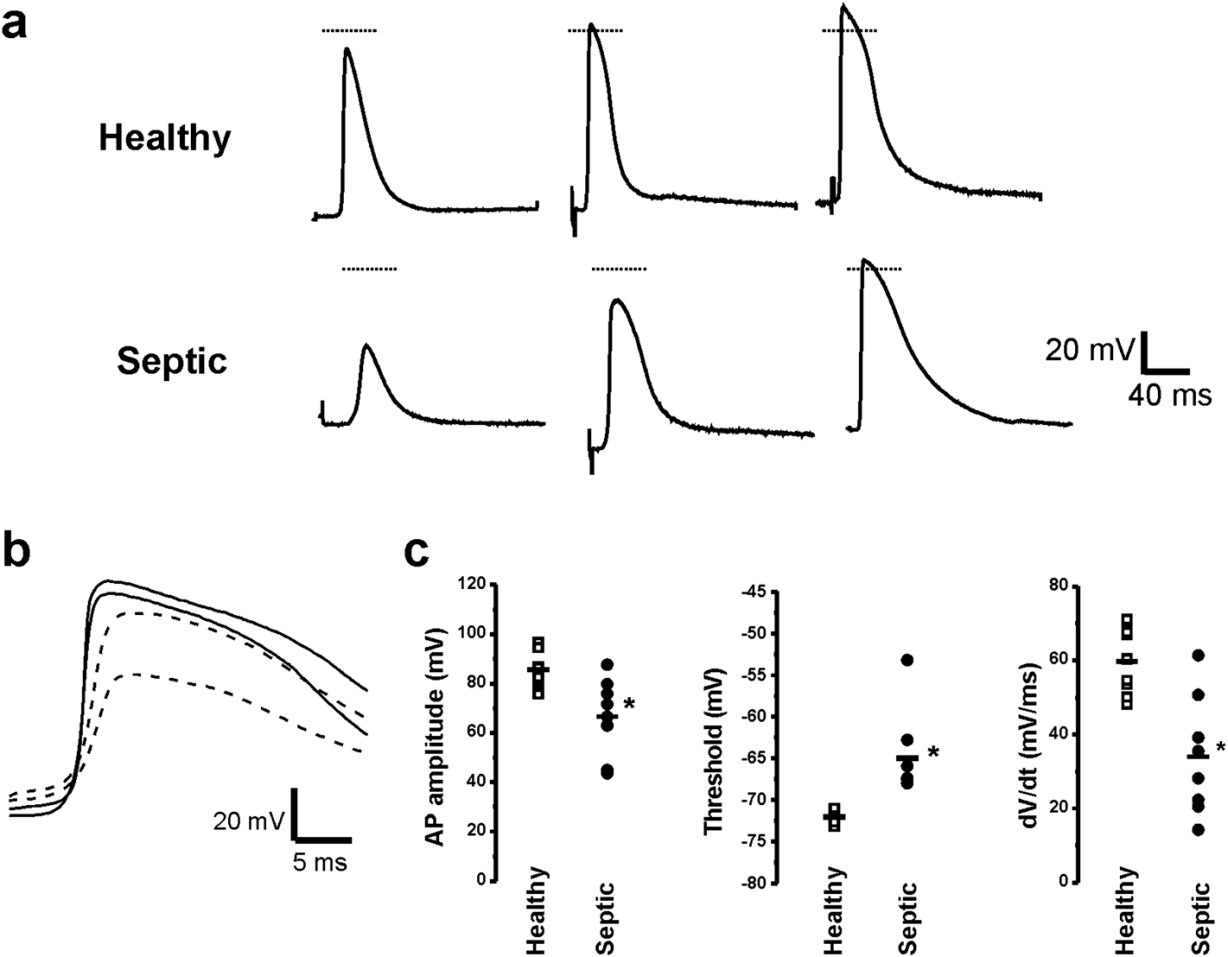
**Changes in action potentials suggest reduced Na current underlies reduced excitability. (a)** Three representative papillary muscle axon action potentials from healthy and septic rats are shown to demonstrate the range of amplitudes in each group. While the largest action potentials from papillary muscle in septic rats were normal in amplitude, only 35% of action potentials overshot 0 mV; whereas in healthy papillary muscles, 73% overshot 0 mV. In most traces, the stimulus artifact can be appreciated prior to the onset of the action potential. The horizontal line represents 0 mV. **(b)** Superimposed on an expanded timescale are two action potentials from healthy rats (solid lines) and two action potentials from septic rats (dashed lines). The action potentials from septic rats have reduced amplitudes and rate of rise, but are similar in duration to those from healthy rats. **(c)** Scatter plots of animal means of action potential characteristics for eleven healthy and eight septic rats. Horizontal lines represent the mean for each group. *P <0.05. AP, action potential; dV/dt, action potential rate of rise.

To directly study excitability and contractility of the heart, left ventricular papillary muscle was taken from control rats and rats killed 24 hours after induction of sepsis by cecal ligation and puncture. The excised muscle was paced at 1 Hz using an extracellular bipolar electrode and action potential characteristics were measured with sharp electrodes. In muscle from septic rats, action potentials were reduced in amplitude by 18.9 mV, threshold was elevated by 7 mV and rate of rise was decreased by 57% (Figure 1, Table 1). Depolarization during cardiac action potentials is caused by opening of both Na and Ca channels. Na channels open more rapidly and are primarily responsible for early attributes of the action potential, including threshold and maximal rate of rise (dV/dt) [25,26]. Changes in these properties suggest reduction in Na current in papillary muscle is the primary mechanism underlying reduction of excitability.

**Table 1:** Characteristics of action potentials

	**V**_**rest **_**(mV)**	**APA (mV)**	**AP threshold (mV)**	**dV/dt (mV/ms)**	**APD 50 (ms)**	**Number of rats studied (number of fibers)**
Healthy	−79.9 ± 1.0	85.5 ± 1.8	−72.0 ± 0.5	59.6 ± 2.4	32.7 ± 2.5	11 (84)
Septic	−78.3 ± 0.6	66.6 ± 5.6*	−65.0 ± 1.8*	34.0 ± 5.7*	29.1 ± 3.2	8 (108)
TTX Studies						
Pre-TTX	−77.5 ± 1.3	88.5 ± 4.2	−67.9 ± 2.5	57.1 ± 5.1	35.3 ± 3.9	3 (14)
Post-TTX	−79.0 ± 2.4	69.1 ± 7.2	−54.6 ± 4.0*	22.1 ± 8.1*	35.0 ± 0.5	3 (21)
Nifedipine studies						
Pre-nifedipine	−82.5 ± 2.3	89.2 ± 2.6	−70.5 ± 4.6	64.5 ± 5.0	38.5 ± 5.2	3 (15)
Post-nifedipine	−85.2 ± 0.6	87.3 ± 2.0	−70.5 ± 2.7	52.4 ± 2.8*	34.3 ± 3.8	3 (22)

V_rest_, resting potential; APA, action potential amplitude; AP threshold, action potential threshold; dV/dt, action potential rate of rise; APD 50, action potential duration at 50% of the maximal amplitude. In healthy and septic studies *P <0.05 septic vs. healthy (unpaired *t* test). In TTX and nifedipine studies *P <0.05 pretreatment vs. posttreatment (paired *t* test).

To determine the effects of selective reduction in Na current on action potential characteristics from healthy rats we applied the specific Na channel blocker tetrodotoxin (TTX) [27] to reduce Na current. The Kd of TTX for cardiac Na channels (Na_v_1.5) is close to 2 μM [28,29], such that 5 μM TTX blocks close to 70% of Na channels. Partial block of Na channels with 5 μM TTX reduced action potential amplitude, elevated threshold and reduced the maximal rate of action potential rise (Table 1). The changes were similar in magnitude to those seen in the most severely affected papillary muscles from septic rats.

A number of ion channels in addition to Na channels determine the characteristics of cardiac action potentials. These include non-voltage gated K channels involved in baseline properties such as membrane potential and specific membrane resistance as well as voltage gated K and Ca channels that contribute to action potential duration [30]. To determine the contribution of these other channels we measured passive membrane properties, action potential duration, and we pharmacologically blocked L-type Ca channels. Sepsis did not induce changes in passive membrane properties and there was no significant depolarization of the resting potential (Table 1). The slight decrease in membrane resistance as estimated by membrane time constant did not reach significance and was not of a magnitude that would significantly reduce excitability (0.90 ± 0.12 ms in healthy vs. 0.78 ± 0.05 ms in septic, *P* = 0.42, n = 3 rats for each group). In addition, the slight decrease in action potential duration was not statistically significant (*P* = 0.38, Table 1). The lack of changes in passive membrane properties and action potential duration suggest sepsis does not trigger dramatic changes in K or Ca currents. To confirm that reduction in Ca current could not account for the reduction in excitability induced by sepsis we measured action potentials in papillary muscle before and after application of 10 μM of the L-type Ca channel blocker nifedipine. Despite its significant effect on contractility (see below), nifedipine had no significant effect on either action potential amplitude or threshold, and triggered only a modest reduction of dV/dt (Table 1). Taken together, these data suggest sepsis-induced reduction in excitability is primarily due to a reduction in Na current rather than changes in either K or Ca currents.

**Figure 2:**
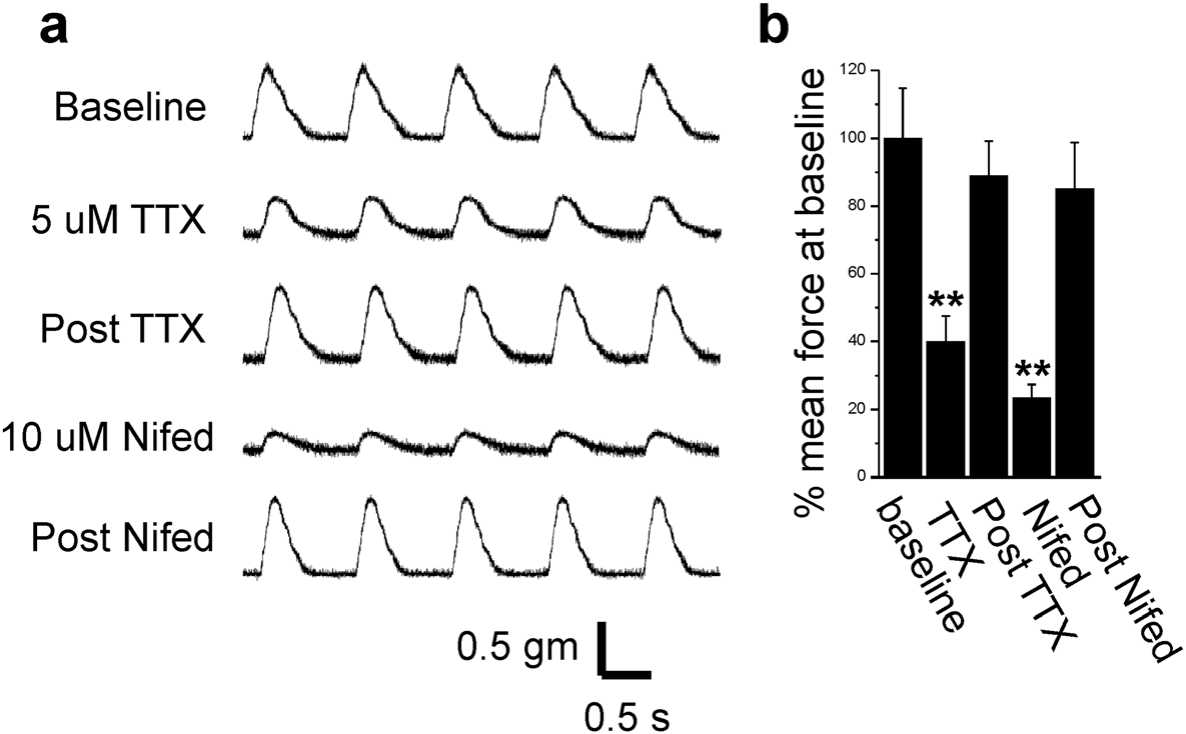
**Block of sodium channels triggers significant reduction in contractility. (a)** Shown are force traces from a papillary muscle paced at 1 Hz before and after application of tetrodotoxin (TTX) and nifedipine. After each drug is washed out, contractility returns to baseline. **(b)** Shown is a bar graph showing the mean force at baseline, following TTX, washout of TTX (post TTX), following nifedipine, and washout of nifedipine (post Nifed) (n = 3 rats). **P <0.01.

While reduction of cardiac excitability during sepsis is of interest, the most clinically relevant problem is reduction of contractility. When *ex vivo* contractility of papillary muscle from septic rats was measured, it was reduced by greater than 70% (papillary muscle force = 0.42 ± 0.08 grams in controls vs. 0.12 ± 0.02 grams in septic rats, *P* <0.05, n = 3 rats). We wished to determine whether reduction in Na current could be a contributor to the reduced contractility induced by sepsis. We measured force generation in papillary muscle from healthy rats before and after application of 5 μM TTX. Partial block of Na channels with 5 μM TTX reduced cardiac contractility by 60% (Figure 2) and was reversible following a 15 minute wash. This suggests that the reduction in Na current induced by sepsis could be an important contributor to reduction in contractility. To determine the importance of Na current relative to L-type Ca current in regulation of contractility, nifedipine was applied. Application of 10 μM nifedipine reduced cardiac contractility by 75% (Figure 2). Washout of nifedipine was not as rapid as washout of TTX; but with a 1 to 2 hour wash, the effect of nifedipine on contractility was largely reversed. Our data suggest reduction of Na current triggers a substantial reduction in contractility that is only slightly less than the effect of reducing L-type Ca current.

## Discussion

### Downregulation of excitability during critical illness

We propose reduced excitability of electrically active tissues is a unifying theme that accounts for the failure of a number of organ systems during sepsis and critical illness. Reduced excitability of peripheral nerve and skeletal muscle has been demonstrated in both patients and animal models of critical illness [1-4,6,7]. We recently demonstrated reduced excitability of neurons within the central nervous system in a rat model of sepsis [8]. If a similar reduction in excitability occurs in the brain of patients it could account for septic encephalopathy. The current study further supports our hypothesis by demonstrating reduced cardiac excitability in a rat model of sepsis.

In both peripheral nerve and skeletal muscle the mechanism underlying reduced excitability is reduction in Na current. Evidence for reduction of Na current in patients comes from studies demonstrating slowing of muscle fiber conduction velocity and an increase in the relative refractory period of both nerve and muscle [3,4,6]. In rats, more direct studies have demonstrated increased inactivation of Na channels as the mechanism underlying reduced excitability of both nerve and muscle during sepsis [7,11]. The current study suggests Na current may also be reduced in the heart. In the current study we did not find evidence to suggest large changes in either Ca channel or K channel function. This raises the possibility that the reduction in Na current is due to a relatively specific effect on Na channels rather than a generalized effect on all ion channels.

There is evidence to suggest that Na current is especially sensitive to cell sickness/injury. In studies of Na current in intact skeletal muscle *ex vivo*, we found minor cell injury during impalement triggers a dramatic hyperpolarized shift in the voltage dependence of the Na current as well as a reduction in maximal current density [20]. Similar shifts have been suggested to occur in injured axons [31,32]. As different Na channel isoforms are present in muscle and nerve, these data suggest that multiple Na channel isoforms may react to cell injury with a hyperpolarized shift in the voltage dependence of activation and inactivation. In injured cells with a depolarized resting potential, a hyperpolarized shift in the voltage dependence of inactivation increases channel inactivation and thus reduces Na current.

What function would be served by down regulation of Na current during cell injury/illness? In heart it has been shown that there is overlap of Na channel activation and inactivation curves such that a Na ‘window’ current could be triggered with depolarization of the resting potential, as would occur during cell injury [33]. This current would further depolarize the cell, and overload it with Na to trigger an elevation of intracellular Ca due to reverse operation of the Na-Ca exchanger [33,34]. Elevated intracellular Ca could then trigger cell death. One way to prevent this cascade of events is to shut down Na current so that cell depolarization, subsequent Na overload, and Ca overload are limited. Shutting down Na current has the cost of reducing excitability and thus temporarily worsening cell function, but might promote cell survival. Reduction of Na current by the cell might serve a similar purpose to the use of barbituate coma in patients: by decreasing metabolic demand during a period of cell stress it might increase cell survival.

### The relationship between reduced cardiac excitability and contractility during sepsis

It is well established that during sepsis there is reduction in cardiac contractility [13-16]. An important contributor to the reduction in contractility is reduction in the action potential-induced Ca transient {Zhong, 1997 #5497}, [35-39]. However, the mechanism underlying the reduction in Ca transient is not well established. Could reduction in Na current contribute to the sepsis-induced reduction in Ca transient and thus contribute to reduction in cardiac contractility?

As intracellular Na does not play a direct role in excitation contraction coupling, reduction in Na current is likely having its effect on contractility via an effect on levels of intracellular Ca. There are several ways reduction in the density of functional Na channels might lessen the action potential Ca transient. The first way is through a reduction in the number of Ca channels that open during the action potential. When Na current is reduced, there is reduction in the peak depolarization reached during the action potential. Reduction in depolarization will reduce Ca channel opening and thus reduce Ca entry. One way this might occur is through shortening of the action potential such that Ca entry is reduced [40].

Alternatively, reduction in Na channels might reduce propagation of action potentials into the t tubules such that cardiac contractility is reduced [41]. Another way is through a direct effect on Ca entry as it has been demonstrated there is Ca entry through Na channels in rat ventricular myocytes [42]. Finally, it has been found that Na entry during the action potential is essential for efficient triggering of SR Ca release [43-45]. The effect is proposed to be mediated by temporary reverse operation of the Na-Ca exchanger, which uses Na entry during the action potential to prime the dyadic cleft with Ca [34,43-45]. These mechanisms may explain the dramatic reduction in contractility we found following partial block of Na channels in healthy papillary muscle. We conclude reduction in cardiac Na current during sepsis may be an important contributor to reduction in contractility.

There are several limitations to this study. (1) We used a rat model of sepsis, which may not faithfully mimic conditions in patients with critical illness. (2) As we used intact, beating, papillary muscle freshly isolated from rats, it was not possible to perform patch clamp studies to directly measure Na and Ca currents. Such studies would necessitate use of *in vitro* models of sepsis using cultured cardiac myocytes. A first step would be to determine whether the finding of reduced cardiac excitability can be recreated in a reduced system *in vitro*. (3) We did not attempt to determine the sodium channel subtype that is affected by sepsis. It has been reported that the heart expresses multiple subtypes of sodium channels that may play a role in contractility [41].

## Conclusions

The present findings indicate that in a rat model of sepsis there is a reduction in cardiac excitability that appears to be due to reduction in Na current. Partial block of Na channels greatly reduced contractility in healthy heart muscle. Together these data suggest that reduction of Na current during sepsis may be a significant contributor to reduction of cardiac contractility. Determining the mechanism underlying reduction in Na current might allow for therapy to improve cardiac contractility during sepsis.

## Key messages

•Cardiac excitability is reduced during sepsis in rats.

•The reduction in excitability appears to be primarily due to reduction of sodium current.

•The reduction in sodium current may be sufficient to explain most of the reduction in cardiac contractility during sepsis.

•Reduced excitability of electrically active tissues may be a unifying theme that accounts for the failure of a number of organ systems during sepsis and critical illness.

## Competing interests

The authors have no competing interests to declare.

## Authors’ contributions

MMR and KLE designed the study. AK performed the experiments and analyzed the data. AK, KLE and MMR drafted the manuscript. All authors read and approved the final manuscript.

## Authors’ information

AK: graduate student, Wright State University. KLE: PhD, Associate Dean of the College of Science and Math, Associate Professor, Department of Neuroscience Cell Biology and Physiology. MMR: MD/PhD, Professor, Department of Neuroscience Cell Biology and Physiology.
